# Pleuroperitoneal Leak Complicating Peritoneal Dialysis: A Case Series

**DOI:** 10.4061/2011/526753

**Published:** 2011-08-17

**Authors:** C. Kennedy, C. McCarthy, S. Alken, J. McWilliams, R. k. Morgan, M. Denton, P. J. Conlon, C. Magee

**Affiliations:** ^1^Department of Nephrology, Beaumont Hospital, Dublin 9, Ireland; ^2^Department of Respiratory Medicine, Beaumont Hospital, Dublin 9, Ireland

## Abstract

Pressure related complications such as abdominal wall hernias occur with relative frequency in patients on peritoneal dialysis. Less frequently, a transudative pleural effusion containing dialysate can develop. This phenomenon appears to be due to increased intra-abdominal pressure in the setting of congenital or acquired diaphragmatic defects. We report three cases of pleuroperitoneal leak that occurred within a nine-month period at our institution. We review the literature on this topic, and discuss management options. The pleural effusion resolved in one patient following drainage of the peritoneum and a switch to haemodialysis. One patient required emergency thoracocentesis. The third patient developed a complex effusion requiring surgical intervention. The three cases highlight the variability of this condition in terms of timing, symptoms and management. The diagnosis of a pleuroperitoneal leak is an important one as it is managed very differently to most transudative pleural effusions seen in this patient population. Surgical repair may be necessary in those patients who wish to resume peritoneal dialysis, or in those patients with complex effusions. Pleuroperitoneal leak should be considered in the differential diagnosis of a pleural effusion, particularly a right-sided effusion, in a patient on peritoneal dialysis.

## 1. Introduction

Peritoneal dialysis (PD) is a well-established means of renal replacement therapy. A Tenckhoff catheter is electively inserted into the peritoneal cavity before PD starts. After a healing period of at least two weeks, PD training begins. In our centre, 500 ml volumes are used for the first two days of training and titrated upwards, based on body surface area, over a two-week period. 

In our centre, there are 35–40 patients on PD at any one time. Approximately 2 patients join our program per month, and a similar number exit the program due to transplantation, switch to haemodialysis, or death. Patients starting PD in our unit use standard dextrose-based, lactate-buffered dialysate. If infusion pain is a problem, bicarbonate/lactate buffered dialysate is used instead. A daytime icodextrin-based dialysate dwell is often required in patients with little residual renal function for extra ultrafiltration. Icodextrin is a hyperosmolar glucose polymer. 

The main complications of PD are either infectious, such as peritonitis and exit site infections, or pressure related, such as abdominal wall hernias and gastrooesophageal reflux. Less frequently, a pleural effusion containing dialysate can develop. This phenomenon appears to be due to increased intraabdominal pressure in the setting of congenital or acquired diaphragmatic defects. 

The incidence rate of pleuroperitoneal leak development is thought to be less than 2% in newcomers to peritoneal dialysis [1]. We report three cases of pleuroperitoneal leak that occurred within a nine-month period at our institution. This corresponded to a 12% incidence rate amongst newcomers to PD in that calendar year (25 newcomers). Prior to that, there had been no cases in our department for over 10 years [2]. We review the literature on this topic and discuss management options. 

## 2. Case 1

A 35-year-old Philipino female presented to our unit with advanced chronic kidney disease, secondary to medullary cystic kidney disease, which was diagnosed many years earlier in the Philipines. She had no significant heart disease. One year later, she was approaching end-stage kidney disease and a Tenckhoff catheter was inserted. Training for PD began four weeks later, using standard dextrose-based solutions. 

**Figure 1:**
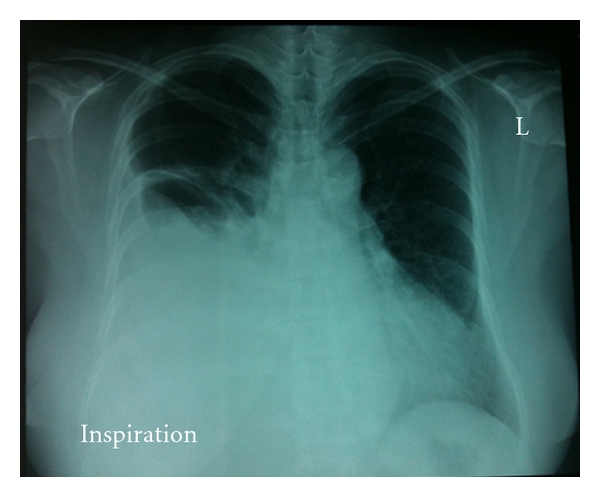
Chest radiograph at presentation.

She presented after four days of PD training with dyspnoea. She was otherwise well and was afebrile. Blood tests and ECG were unchanged. She had clinical evidence of a large, rightsided pleural effusion. This was confirmed on a chest radiograph (Figure 1). A diagnostic aspirate was performed and yielded serous fluid. The pleural fluid biochemistry was consistent with a transudative process (Table 1). The high-pleural-fluid-serum-glucose ratio confirmed the clinical suspicion of a pleuroperitoneal leak. 

**Figure 2:**
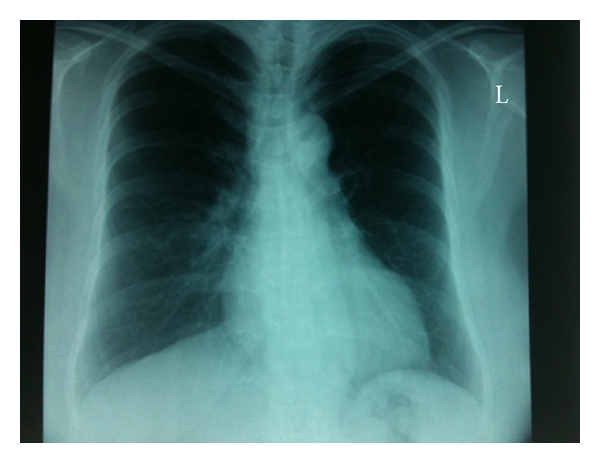
Follow-up chest radiograph.

The pleural effusion resolved over a number of days with conservative management and the maintenance of a dry peritoneal cavity. A follow-up chest radiograph was normal (Figure 2). In accordance with patient preference, PD was discontinued. Haemodialysis (HD) access was established and HD is ongoing one year later.

## 3. Case 2

A 38-year-old Romanian female was admitted acutely with symptomatic uraemia. Her background was significant for minimal change disease, diagnosed in 2001 in Romania. She had received two courses of heavy immunosuppression for this and was then lost to follow up. She had no known heart disease. Her renal ultrasound showed small, shrunken kidneys, which confirmed the suspicion of advanced chronic kidney disease.

Acute HD was initiated but, as the patient's preference was for PD, a Tenckhoff catheter was inserted. Five weeks later, PD training began. Her target PD prescription consisted of four cycles of two litre exchanges with standard dextrose-based standard solution. She was also prescribed one icodextrin-based dwell by day. 

After six days of PD training, she presented with dyspnoea that was exacerbated by infusing dialysate. She had no other systemic symptoms. She was seven kg above her dry weight and had clinical evidence of a rightsided pleural effusion and pedal oedema. Her electrocardiogram (ECG) and routine blood tests were unchanged. As she appeared volume overloaded, she had three litres of isolated ultrafiltration, using her central venous catheter. Her diuretic regime was maximized and she was discharged. 

Despite these interventions, she presented several days later with worsening dyspnoea. A chest radiograph confirmed a large rightsided pleural effusion. Again, she had 3 litres of isolated ultrafiltration. With this, she developed bad cramping. Therapeutic thoracocentesis was performed, with the removal of 1.2 litres of serous fluid. This led to marked clinical and radiological resolution of the pleural effusion. 

Although the initial presumed diagnosis was volume overload, the high pleural fluid glucose relative to the serum glucose confirmed the presence of a pleuroperitoneal leak (Table 2). In accordance with patient preference, PD was discontinued and the Tenckhoff catheter was removed. HD is ongoing without complication.

## 4. Case 3

A twenty-four-year-old Irish male with a background history of congenital deafness, intellectual impairment and repair of a posterior urethral valve initially presented to the paediatric nephrology services with nephrotic syndrome. A renal biopsy revealed secondary focal segmental glomerulosclerosis and significant tubuleinterstitial fibrosis. Following introduction of Renin-Angiotensin-Aldosterone system blockade, his proteinuria was controlled. 

Despite this, his chronic kidney disease progressed. Ten years later, he was approaching end-stage kidney disease. A Tenckhoff catheter was inserted without complication. Six weeks later, PD training was initiated. A bicarbonate/lactate buffered solution was used to avoid infusion pain, which he would be unable to verbalise. The following months were complicated by dialysis-associated pericarditis that resolved with intensive haemodialysis for a number of weeks. His baseline PD prescription consisted of seven cycles of two litre exchanges with 1.36% dextrose solution. He was also prescribed an icodextrin-based daytime dwell. 

Five months later, he was admitted with clinical and radiological evidence of a large, rightsided pleural effusion with pleural thickening. The exact duration of the effusion was unclear as he was unable to verbalise symptoms. It was thought to have developed over weeks given the apparent absence of symptoms and the degree of pleural thickening. A chest radiograph four months earlier showed normal lung fields. 

He was afebrile and systemically well. His serum biochemistry and haematology were unchanged and his inflammatory markers were not raised. ECG and echo were normal. A diagnostic pleural aspirate was performed under ultrasound guidance. This yielded serous fluid and also identified numerous loculations within the collection. 

The pleural fluid biochemistry was atypical for that seen with pleuroperitoneal leak (Table 3). However, as the patient was so well clinically, and numerous pleural fluid cultures were sterile, it was felt that the effusion was not related to infection. It was also felt that the effusion was not due to volume overload given the degree of loculation within the effusion and the absence of other clinical signs of volume overload. The low pleural fluid glucose and high lactate dehydrogenase (LDH) were difficult to interpret as the sampled fluid was walled off within a loculation, probably for many weeks. A clinical diagnosis of pleuroperitoneal leak was made and PD was discontinued.

The effusion did not resolve with drainage of the peritoneal cavity, and a chest drain was inserted. This drained minimal serous fluid due to the highly loculated nature of the effusion. A second drain was inserted and intrapleural alteplase administered. This drain became dislodged and was replaced by a third drain with further intrapleural alteplase administration. At this point, however, a chest radiograph confirmed persistence of the large, loculated effusion with a small pneumothorax and a thick pleural rind. 

Video-assisted thorascopic surgery with decortication was performed. A rapid postoperative recovery ensued and the postoperative chest drains were removed without event. A chest radiograph six weeks later showed a well-expanded right lung, without effusion or pleural abnormality. HD is ongoing without complication.

## 5. Discussion

The first description of a pleuroperitoneal leak causing a pleural effusion in a PD patient was in 1967 [3]. Since then, several reports have estimated the incidence of this complication; the largest report estimated an incidence of 1.6% [1]. 

It is thought that congenital or acquired communications between the pleura and peritoneum underpin this problem. This had been demonstrated both in scintigraphy [4] and in autopsy specimens with localized absence of diaphragmatic muscle fibres [5]. The raised intraabdominal pressure with dialysate infusion, in a patient with such a communication, promotes the translocation of dialysate into the pleural space. 

An increased incidence of pleuroperitoneal leak is seen in patients with polycystic kidney disease [6]. This is probably related to the increased intraabdominal pressure in these patients and, therefore, higher pleuroperitoneal pressure gradients [6]. There is also a generalized connective tissue weakness in this condition that may contribute to inherent diaphragmatic weakness [6]. Patients with previous peritonitis are also at higher risk of developing this condition. This is probably due to a weakening of diaphragmatic tissue during peritonitis [7]. 

The timing of this complication varies from days to years after the initiation of PD. Half of cases occur within one month of starting PD [3]; these probably represent patients with congenital diaphragmatic defects. Those cases that occur later probably represent those with acquired diaphragmatic defects. Patients typically present with dyspnoea, although a significant proportion are asymptomatic [3]. Clinical examination reveals a pleural effusion that is usually rightsided [3]. Presumably the preponderance of rightsided cases is due to diaphragmatic protection by the heart on the left. 

Pleural effusions are broadly divided into transudative and exudative effusions. Transudative effusions develop when systemic factors affect the pleural Starling forces, as seen, for example, in congestive cardiac failure. Exudative effusions develop when local factors influence the formation of pleural fluid, as seen in malignant effusions. Light's criteria are applied to the pleural and serum biochemistry to make the distinction between a transudate and an exudate [8]. An effusion is an exudate if the pleural-fluid-to-serum-protein ratio is >0.5 or the pleural-fluid-to-serum-LDH ratio is >0.6 [8]. Pleuroperitoneal leaks typically cause transudative effusions with low LDH and low cell count [5]. 

The differential diagnosis of a pleural effusion in a dialysis patient is extensive. Transudative pleural effusions due to volume overload or congestive cardiac failure are relatively common in this population. In PD patients, such effusions would typically be managed by increasing the dialysate volume and tonicity to increase ultrafiltration. However, this can exacerbate the problem if the effusion is due to a pleuroperitoneal leak.

This highlights the importance of the pleural fluid glucose measurement. In the absence of loculation, a high pleural fluid to serum glucose concentration gradient is a very sensitive and specific test for diagnosing a pleuroperitoneal leak [9]. The ratio of pleural fluid to serum glucose is dynamic, and varies depending on the type of fluid instilled, the volume and the contact time. However, a positive gradient is highly suggestive of a pleuroperitoneal leak [9]. This is due to the presence of hypertonic, usually dextrose-based, dialysate in the pleural space—a “sweet hydrothorax” [10]. Atypical biochemistry can be seen when the fluid has been in the pleural cavity for a prolonged period and has been partially reabsorbed by lymphatics, as seen in Case 3.

Other diagnostic tests are less practical and include Technetium-99m labelled peritoneal scintigraphy [4] and computed tomography (CT) with intraperitoneal dye [11].

In terms of management, emergency large volume thoracocentesis is occasionally required [3]. However, most cases of pleuroperitoneal leak are initially managed by drainage of the peritoneal cavity [3]. Temporary HD may be required, especially for those with minimal residual renal function. Many patients choose to remain on HD in the long term and, therefore, formal repair of the diaphragm is not required.

For those that wish to return to PD, there are a number of management options. One group advocates early diaphragmatic repair and continuation of peritoneal dialysis [12]. In this case, thorascopic diaphragmatic repair was performed using absorbable polyglycolic acid felt, and fibrin glue [12]. 

Most advocate temporary discontinuation of PD. This allows resolution of the effusion and, in some cases, healing of the diaphragmatic defects. A trial of low-volume PD two–six weeks later is successful in a significant proportion of cases [13]. 

Recurrent hydrothorax necessitates either a permanent switch to haemodialysis or definitive management of the pleural-peritoneal communication. Chemical pleurodesis via an intercostal drain has been used in several cases with success [13]. Agents used include tetracycline, blood and fibrin glue. This method had relatively high rates of success, with 48% of patients resuming long-term PD after pleurodesis in one large systematic review [13]. 

Video-assisted thorascopic surgery, with direct visualization of the diaphragmatic defect and suture repair, has shown much promise in recent times for the management of this condition [13, 14]. Using this approach, 88% of patients in one study successfully resumed long-term PD without recurrence [13]. 

The three cases described above highlight the variability of this condition in terms of timing, symptoms, and management. Pleuroperitoneal leak should be considered in the differential diagnosis of a pleural effusion, particularly a rightsided effusion, in a patient on peritoneal dialysis. 

## Figures and Tables

**Table 1:** Biochemical results.

	Pleural fluid	Serum
pH	7.64	7.38
Albumin (g/dL)	<15	32
HCO3—(mmol/L)	30.1	28
LDH (iu/L)	<50	474
Glucose (mmol/L)	12.1	5.2
Amylase (iu/L)	<10	40

**Table 2:** Biochemical results.

	Pleural fluid	Serum
pH	7.55	7.37
Albumin (g/dL)	<15	39
HCO3—(mmol/L)	29	32
LDH (iu/L)	<50	379
Glucose (mmol/L)	8.6	6.1

**Table 3:** Biochemical results.

	Pleural fluid	Serum
pH	7.23	7.36
Albumin (g/dL)	28	29
HCO3—(mmol/L)	20	26
LDH (iu/L)	2816	614
Glucose (mmol/L)	<0.6	4
Amylase (iu/L)	<10	32
